# Identification and Quantification of Explosives in Nanolitre Solution Volumes by Raman Spectroscopy in Suspended Core Optical Fibers

**DOI:** 10.3390/s131013163

**Published:** 2013-09-30

**Authors:** Georgios Tsiminis, Fenghong Chu, Stephen C. Warren-Smith, Nigel A. Spooner, Tanya M. Monro

**Affiliations:** 1 Institute for Photonics & Advanced Sensing and School of Chemistry & Physics, the University of Adelaide, Adelaide, South Australia 5005, Australia; E-Mails: stephen.warrensmith@adelaide.edu.au (S.C.W.-S.); nigel.spooner@adelaide.edu.au (N.A.S.); tanya.monro@adelaide.edu.au (T.M.M.); 2 School of Computer Science and Information Technology, Shanghai University of Electric Power, Shanghai 200090, China; E-Mail: chufenghong@siom.ac.cn; 3 Defence Science & Technology Organisation, South Australia 5111, Australia

**Keywords:** explosives detection, fiber sensors, Raman spectroscopy, chemical sensing, microstructured optical fibers

## Abstract

A novel approach for identifying explosive species is reported, using Raman spectroscopy in suspended core optical fibers. Numerical simulations are presented that predict the strength of the observed signal as a function of fiber geometry, with the calculated trends verified experimentally and used to optimize the sensors. This technique is used to identify hydrogen peroxide in water solutions at volumes less than 60 nL and to quantify microgram amounts of material using the solvent's Raman signature as an internal calibration standard. The same system, without further modifications, is also used to detect 1,4-dinitrobenzene, a model molecule for nitrobenzene-based explosives such as 2,4,6-trinitrotoluene (TNT).

## 1. Introduction

Explosives detection is an area of chemical sensing of particular interest as a result of the advent of improvised explosive devices (IEDs) and terrorist activities that cause concerns for national security both home and abroad. As explosive devices move away from incorporating metal shells and parts, the need to detect the actual explosive molecules becomes imperative [1,2]. Amongst the various detection schemes, optics-based detectors show great promise as they allow non-invasive interrogation of species, can be applied across different analyte phases and are based on robust optoelectronics technology. Photoluminescence and chemoluminescence sensors in particular have shown potential for explosives detection, albeit with their own limitations [3]. While photoluminescence-based explosives detection has been shown to enable very low detection limits, false-positives can affect the selectivity of this technique. Chemoluminescence deals with this challenge by using analyte-specific binding sites and reactions to produce the detected optical signal; however this approach requires individual detector preparation depending on the analyte under investigation [4–6].

An alternative set of optical detection techniques are based on Raman and infrared (IR) spectroscopy, whereby the interactions between excitation light and molecular species result in unique, fingerprint-like spectra that can be compared against tables of materials to uniquely identify the explosive molecules [7–10]. However, these techniques suffer from relatively low signal intensities compared to luminescence detection [11], thus requiring additional signal enhancement techniques such as surface-enhanced Raman scattering (SERS) [12].

Across all the optical detection techniques, optical fibers have been successfully used in sensing chemical and explosive species due to their robustness, ability to access difficult-to-reach areas and their immunity to electromagnetic interference that allows them to separate the detection area from the controlling electronics [13,14]. Suspended-core optical fibers are a category of microstructured optical fibers in which a light-guiding core is suspended inside a capillary-like fiber jacket by a number of struts, essentially creating a micro- or nano-wire waveguide suspended inside a protective shell [15,16]. These small core dimensions result in a significant overlap of the guided light with any liquid or gas that is loaded into the holes in the fiber, which in turn creates a response signal along the entire length of the fiber that is subsequently waveguided by the core to a detector [17]. Sensing platforms based on suspended-core fibers are of particular interest to small-volume chemical and biological sensing as the signal generated by the analyte is integrated along the length of the fiber, resulting in low detection limits while their filling volumes are typically in the range of 10 s of nanoliters [17,18]. Prior experimental demonstrations of Raman and surface-enhanced Raman sensing in microstructured fibers have been focused on short lengths of hollow- and solid-core photonic crystal optical fibers [19–24]. Suspended-core fiber Raman sensors have been proposed [25], but surface functionalization to achieve SERS effect is required to observe any signal, increasing the sensor complexity and preparation time [26].

In this work, we use suspended-core optical fibers as an active dip sensor platform for Raman sensing of explosives without need for modifications when switching between species. This platform is used for detection of hydrogen peroxide (H_2_O_2_), a material used in preparing homemade explosives that lacks the nitroaromatic groups that 2,4,6-trinitrotoluene (TNT)-based explosives use and therefore is more difficult to detect with traditional techniques based on specific chemical group interactions [27,28]. Numerical modeling is performed for these sensors that includes coupling of both the excitation and Raman fields to high order optical modes as a guide for fiber core radius selection, guiding the selection of a 0.85 μm radius fiber as the most effective Raman sensor. The small sampling volume (60 nL), combined with long interaction lengths inside the fiber, result in detected, quantifiable amounts of less than 1 μg of hydrogen peroxide in aqueous solutions. The same unmodified system is shown to also detect comparable amounts of 1,4-dinitrobenzene (DNB), a substitute for TNT-like molecules, highlighting the flexibility and unique identification capability of this technique.

## 2. Experimental Section

**Figure 1.**
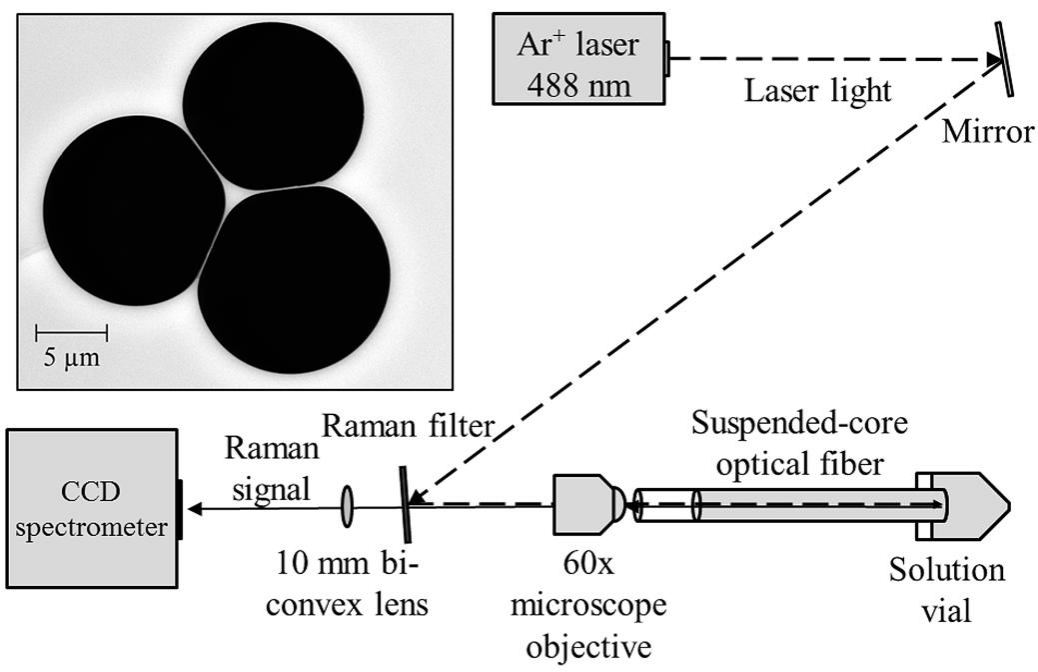
Experimental setup used for Raman measurements of explosives in suspended-core optical fibers. The reflection angle off the Raman filter has been exaggerated for clarity. The inset shows a scanning electron microscope image of the silica fiber used in the experiments.

The experimental setup used in these experiments is shown in Figure 1. Continuous wave light from an Ar^+^ laser (Melles Griot Series 43) at 488 nm is reflected off a Raman filter (Semrock 488 nm long-pass RazorEdge Ultrasteep) and is coupled into a suspended core fiber using a 60× microscope objective (focal spot radius 0.85 μm) to deliver 70 mW to the fiber front face, with 22 mW measured at the far end of the fiber due to coupling efficiency and fiber loss (31% throughput). Initially a 20 cm length of suspended core fiber was used, made in-house from silica glass (F300, Heraeus), with a core radius of 0.85 μm (based on the radius of a circle that has the same area as a triangle that completely fits within the core region) and a hole radius of 6.3 μm, also shown in Figure 1. The far end of the fiber is dipped into a glass vial containing an aqueous solution of hydrogen peroxide and the fiber is allowed to fill by capillary force action to a total filled length of 16 cm to avoid droplet formation at the coupling end, resulting in a total sampling volume of 60 nL. The Raman signal from the fiber is collected in backscattering mode through the Raman filter and is analyzed using a cooled-CCD spectrometer (Horiba Jobin Yvon iHR320) with an 1800 lines/mm holographic grating, resulting in a spectral resolution of 0.03 nm.

One of the factors that can affect the behavior of a suspended core fiber used for Raman sensing is the Raman signal originating in the glass core itself. The degree to which this occurs depends on the core material, the molecules detected, and the geometry of the fiber. The excitation light travelling down the fiber core will induce a Raman signal from the glass that will be overlaid on the signal created by the analyte molecules around the core. Silica glass is known to have a Raman signature originating in the vibrational movements of the Si–O lattice [29].

**Figure 2.**
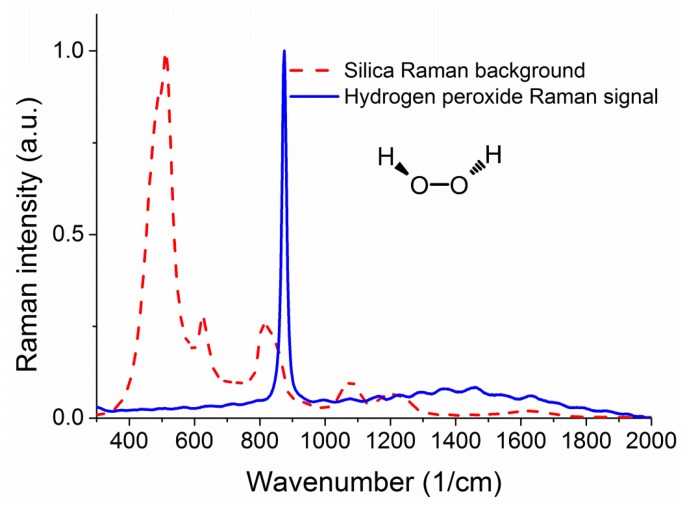
Raman spectra of silica glass (red dashed line) and hydrogen peroxide (blue solid line). The spectra have been normalized to their maximum value for comparison.

Figure 2 shows a measured Raman signature of the unfilled silica suspended core fiber under 488 nm excitation from an Ar^+^ laser, where the units for the x axis are inverse centimeters, expressing the difference in energy between the laser light and the observed spectral features. The same figure also shows the Raman signature of hydrogen peroxide for comparison, taken using an Agiltron H-PeakSeeker Pro-785 desktop Raman spectrometer.

The main peak from hydrogen peroxide appears at 876 cm^−1^ and is attributed to the O–O stretching mode [30,31]. As seen in the graph there is a significant degree of overlap between the two spectra in the 800 to 900 cm^−1^ region, meaning that the results from the experiments will be mostly a function of the peroxide-to-silica (signal-to-background) ratio of Raman signals, rather than the net Raman signal of hydrogen peroxide. To this end it is essential to study the effects of changing the fiber sensor parameters that affect the ratio between the background Raman signal from the core and the Raman signal from the analyte sample in the holes surrounding the core. For a given fiber core material, the strongest parameter that can change this ratio is the size of the light-guiding core and therefore numerical simulations are required to determine the optical fiber with the best sensing performance.

## 3. Results and Discussion

### 3.1. Numerical Modeling of Suspended Core Optical Fibers as Raman Sensors

**Figure 3.**
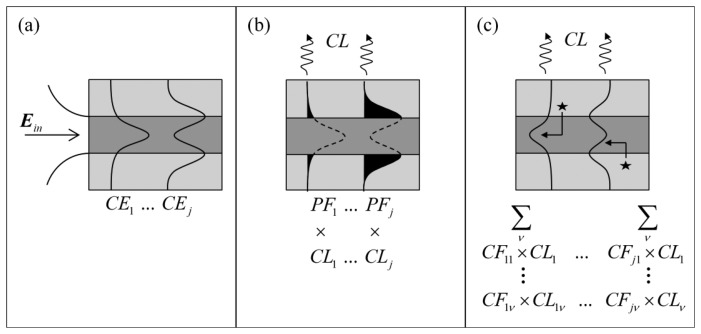
An overview of (**a**) the coupling of excitation light into different fiber core (dark grey) optical modes; (**b**) the power fractions of the optical modes (black shaded area) outside the solid core showing the confinement losses due to the dimensions of the core; (**c**) Raman scattering sites (stars), showing coupling of the Raman signal into different optical modes.

In evanescent field sensing experiments using suspended core fibers, it has been shown that smaller core radii result in enhanced signals when background signals are present [32]. Much like the fluorescence measurements, the signal-to-background ratio between the Raman signals from hydrogen peroxide in the holes of the suspended core fiber and the silica core is a direct result of the geometry of the fiber, which supports a small number of optical modes at the wavelengths and core sizes used here (19 modes for a 1 μm core radius fiber, 67 modes for a 2 μm core radius). In order to guide the fiber design process by identifying which fiber core dimensions will produce the highest signal-to-background ratio for Raman sensing experiments, numerical modeling of the suspended core fibers was performed. The model assumes a circular diameter suspended silica core surrounded by water, the solvent used in the experiments, to investigate the effect of core radius on the performance of the fiber sensor. Initially, excitation light *E_in_* is coupled into different optical modes within the fiber with a given coupling efficiency (*CE_j_*) for each mode, *j*, as shown in Figure 3a and defined in Equation (1) [33].

(1) $$CE_{j}{\left|\left(\frac{2(\int_{\infty }E_{in}\times h_{j}^{*}\cdot \hat{z}dA)(\int_{\infty }e_{j}\times H_{in}^{*}\cdot \hat{z}dA)}{\int_{\infty }E_{in}\times h_{j}^{*}\cdot \hat{z}dA+\int_{\infty }e_{j}\times H_{in}^{*}\cdot \hat{z}dA}\right)\right|}^{2}$$

*E_in_* and *H_in_* are the electric and magnetic field intensities of the input beam, *e_j_* and *h_j_* are the electric and magnetic field distribution of the fiber mode *j* and *z* is the direction of propagation of the mode along the length of the fiber.

The guided light has components both in the core and in the cladding (in this case the solution-filled holes around the core) and therefore the corresponding power fractions *PF_j_* of the guided light for each mode will determine the strength of interaction with the silica core and analyte molecules in the holes respectively [34]. The power fraction for each excitation mode *j* is given by Equation (2) below:

(2) $$PF_{j}=\frac{\int_{x}e_{j}\times h_{j}\cdot \hat{z}dA}{\int_{\infty }e_{j}\times h_{j}\cdot \hat{z}dA}$$

The capture fraction (*CF_jv_*), defined as the fraction of the total Raman scattered signal generated by each excitation mode *j* across the entire area of the hole *H* and collected by each Raman wavelength mode *v*, is given by Equation (3) [34,35]:

(3) $$CF_{jv}=\frac{\lambda_{v}^{2}}{16\pi n_{v}^{R}}{\left(\frac{{ɛ}_{0}}{\mu_{0}}\right)}^{\overset{1}{\;}/\underset{2}{\;}}\frac{\int_{H}s_{j}{\left|e_{v}\right|}^{2}dA}{\left|\int_{H}s_{j}dA|\;|\int_{\infty }s_{v}dA\right|}$$

In Equation (3) *λ* is the Raman wavelength, *n_v_* is the solvent refractive index at the Raman wavelength, *ε_0_* is the electric permittivity of free space, *μ_0_* is the magnetic permeability of free space, and *s_j_* is the component of the pointing vector along the fiber axis (z direction) for the mode *j*. This definition assumes that the Raman scattering is uniform in all directions and has random polarization. Confinement loss (*CL*) is also considered, which becomes important for microstructured optical fibers due to the cladding index and core index being the same and thus the core supports leaky modes [33]. Thus, the capture fraction as a result of a given excitation mode *j* that scatters into all Raman wavelength modes *v* is expressed as Equation (4):

(4) $$CF_{j}=\sum_{v}\left[CF_{jv}\times CL_{v}\right]$$

The figure of merit (*FOM_x_*) for either the Raman generated in the core (*x = C*) or in the holes (*x = H*) is then a function of the power fraction (*PF_j_*) available in each excitation mode and the corresponding coupling efficiency (*CE_j_*), the confinement loss (*CL_j_*) of the mode and finally the capture fraction (*CF_j_*) as defined above, across all excitation and Raman optical modes and is given by Equation (5):

(5) $$FOM_{x}=\sum_{j}\left[CE_{j}^{x}\times PF_{j}^{x}\times CL_{j}^{x}\times CF_{j}^{x}\right]$$

The total figure of merit (*FOM_T_*) for the system, which takes into account both the analyte Raman signal generated in the holes and the background signal generated in the core is given by Equation (6). This parameter is expected to correlate to experimentally observed signal-to-background quantities, thus guiding the choice of fiber:

(6) $$FOM_{T}=FOM_{H}/FOM_{C}$$

**Figure 4.**
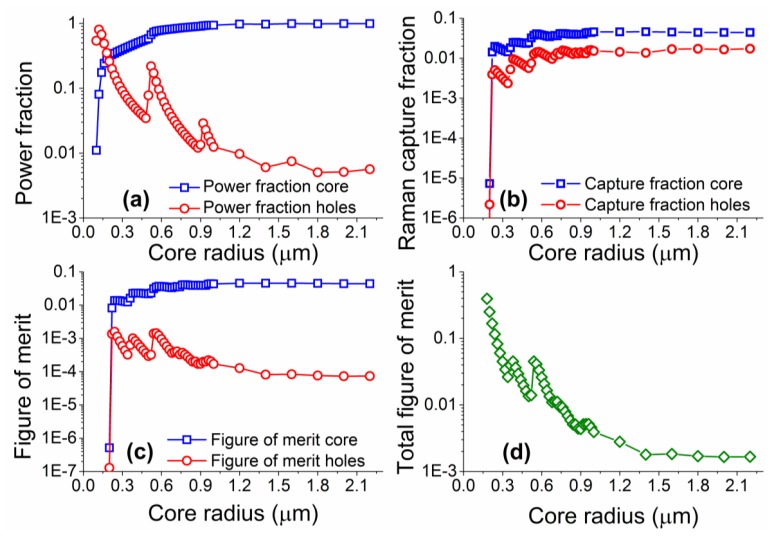
(**a**) Power fraction (including higher order excitation modes); (**b**) Backwards capture fraction; (**c**) Figure of merit for Raman signals originating in the core (squares) and the holes (circles) of a suspended core fiber surrounded by water on a logarithmic scale as a function of the core radius for 488 nm excitation and 510 nm detection; (**d**) Total figure of merit for Raman signal from analytes in the holes around a suspended core over the background Raman signal of the silica core as a function of changing core radius. The lines are guides for the eye.

Numerical simulations were performed for core radii between 0.1 and 2.2 μm, with coupling of both excitation and Raman signals into both the fundamental and higher order modes considered. The model was based on vector solutions of a step-index fiber, based on the work previously developed for fluorescence sensing [16,32,34], but with all excitation modes considered, and the coupling efficiency into these modes defined as given in Equation (1). Note that material and scattering losses were not included in the model as the proximity of the excitation and Raman wavelengths result in practically identical values of material loss at both wavelengths. Figure 4 shows the results from the numerical simulations performed across a range of different core sizes.

The power fraction for the laser excitation follows the trend shown in previous work on suspended core fibers, whereby as the core becomes larger the fraction of light travelling in the holes around the core becomes smaller. As the core increases in size, a larger number of optical modes become available, resulting in apparent jumps in the power fractions guided through the fiber. The figure of merit, as defined here, becomes larger for Raman signal originating in the holes around the core as the core radius is reduced, with the opposite trend for Raman signal from the core of the fiber. Similarly to the power fraction results, deviations from the general trend are observed when more high order modes become available at different core radii. The total figure of merit calculations predict that there is a clear advantage in using small radius suspended cores, as the expected signal-to-background ratio increases with reducing core sizes. The behavior for very small core sizes (smaller than 0.5 μm) differs from that calculated for fluorescence measurements [32] in that there is no drop in the total figure of merit as the core radius becomes exceedingly small. This is due to the close proximity of the excitation and Raman wavelengths (22 nm apart for 488 nm excitation) that results in very similar confinement losses. In reality very small core sizes can exhibit reduced coupling efficiency of the excitation light, increased scattering losses from surface imperfections [36], and are more difficult to handle; our results though clearly show small core radii are better suited for Raman experiments when the silica Raman background overlaps with the analyte Raman signal.

**Figure 5.**
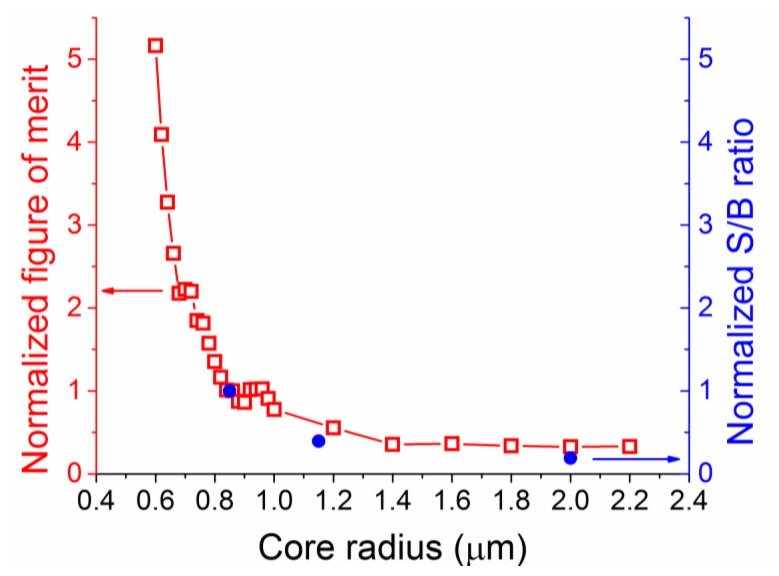
Total figure of merit (squares) and experimental signal-to-background (S/B) ratio (circles), normalized at a core radius of 0.85 μm for ease of comparison, as a function of core radius. The graph also shows the expected improvement in signal-to-background ratio for smaller core radii.

To verify these simulation results, silica suspended core fibers of different core radii (0.85, 1.15 and 2 μm) were fabricated and identical lengths were filled with an aqueous solution of hydrogen peroxide (30% w.t.). As an indication of total energy, the area under the curve in the wavenumber region where the Raman peak for hydrogen peroxide appears (876 cm^−1^) was integrated across its width for both empty and filled fibers, and the ratio between the hydrogen peroxide signal and the silica background was compared against the numerical results for the total figure of merit as a function of the core radius. These two quantities, the experimental signal-to-background ratio and the total figure of merit are not directly comparable in terms of absolute values as the model does not consider the total signal produced by the fiber, but they should be qualitatively comparable. To make a direct comparison between them, both ratios were normalized at their respective values for the minimum experimental core radius of 0.85 μm, as seen in Figure 5. The trend predicted across this range of core radii from the numerical calculations is in good agreement with the observed signal-to-background ratio, verifying the choice of the smallest available silica suspended core for Raman measurements of hydrogen peroxide. The graph also highlights future gains in signal-to-background ratios by fabricating and using even smaller core radius fibers, although the absolute value of enhancement is expected to vary somewhat for smaller size cores [36]. Based on these results the 0.85 μm core radius suspended core fiber was chosen for further experiments and analysis.

### 3.2. Raman Sensing of Explosives in Suspended Core Fibers

**Figure 6.**
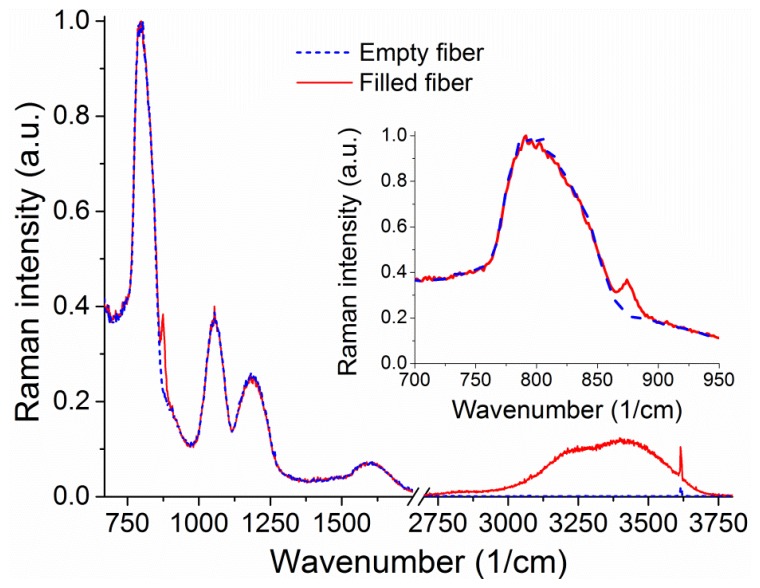
Raman signal collected from an empty silica suspended core fiber (blue, dashed line) and the same fiber filled with a hydrogen peroxide aqueous solution (red, solid line) for 488 nm CW excitation. The region from 2,000 to 2,600 cm^−1^ is not shown as it contains no useful information. The inset shows the region of interest for hydrogen peroxide, between 700 and 950 cm^−1^ for clarity.

The Raman signal from an unfilled 20 cm piece of suspended core silica fiber with a core radius of 0.85 μm can be seen in Figure 6. When the fiber is filled up to 16 cm to avoid droplet formation on the collection end (loading time 4.7 min) with a hydrogen peroxide solution (30% w.t. in water), the sample vial is removed from the end of the fiber. When the fiber is full the water peak is easily observable, from 3,200 to 4,000 cm^−1^, originating in the O–H stretching [37,38]. This broad peak arises from both water and hydrogen peroxide Raman signals and is therefore representative of the total number of OH-containing molecules. The smaller sharp features at 1,050 cm^−1^ and 3,650 cm^−1^ appear for both empty and filled fibers and are likely due to light pollution from parasitic lines of the Argon ion laser used.

**Figure 7.**
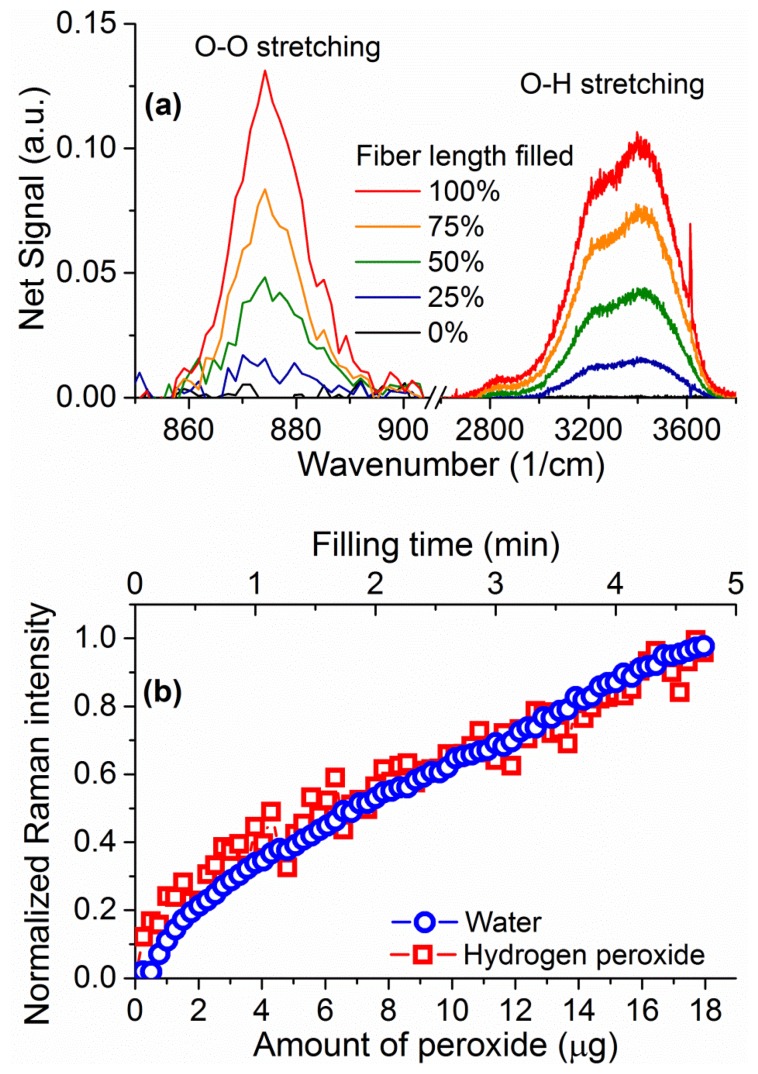
(**a**) Signal evolution for the hydrogen peroxide (876 cm^−1^) and water (3,400 cm^−1^) Raman signals, after subtraction of the glass background signal as a function of the filled length percentage of the fiber; (**b**) Integrated intensity for the hydrogen peroxide (squares) and water (circles) Raman spectra as a function of the amount of hydrogen peroxide inside a suspended core fiber. Both graphs are shown for a 30% w.t. concentration of hydrogen peroxide in water.

Upon closer inspection of the area where the hydrogen peroxide Raman signature is expected, a peak is visible above the silica background at 876 cm^−1^ (inset of Figure 6) corresponding to the hydrogen peroxide O–O stretching Raman peak [29]. The fact that this signal is visible in the absence of surface-enhancing processes is a direct result of the ability of the suspended-core architecture to create, collect, and guide Raman scattered signal throughout the entire length of the fiber. To remove the silica background, the collected spectra are normalized at the first silica peak, which is not expected to change in shape as the silica core is unaffected by the filling process, and the signal for the empty fiber is then subtracted. The resulting spectra for hydrogen peroxide and water are easily distinguishable and their evolution with filling time can be studied, as shown in Figure 7 for 30% w.t. hydrogen peroxide in water. Both signatures increase in intensity as the fiber fills up with hydrogen peroxide solution by capillary force action. As an indication of the total intensity of the Raman scattering for each molecule, the area under the spectral curve is integrated after background subtraction. As the fiber geometry and filling time are well known, this integrated Raman signal can be plotted as a function of the amount of hydrogen peroxide, seen in Figure 7b. This allows the determination of the minimum amount of hydrogen peroxide that gives a clearly identifiable signal detectable by the system as 1 μg, for a 30% w.t. hydrogen peroxide aqueous solution inside a 16 cm piece of fiber, limited by the intensity of the silica Raman background signal.

**Figure 8.**
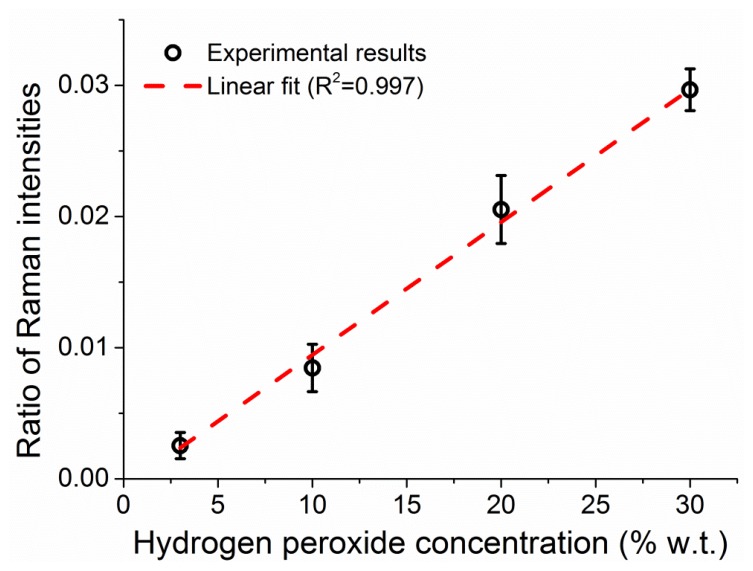
The ratio of the integrated Raman intensities of hydrogen peroxide and water in an aqueous solution inside a suspended core fiber as a function of the peroxide concentration. The dashed line is a linear fit (*R*^2^ = 0.997).

The integrated intensities of the peroxide and water Raman peaks increase in parallel as the fiber fills up, reflecting a proportional increase in the number of molecules for both the hydrogen peroxide and the water. The ratio between the two peak intensities is dictated by the ratio of molecules between the two (*i.e.*, the hydrogen peroxide concentration in water) and the relative intensities of the Raman scattering. By measuring solutions of different concentration of hydrogen peroxide, 3%, 10%, 20%, and 30% w.t., it is possible to correlate the ratio between the two Raman peaks to the weight percentage of hydrogen peroxide in water, as seen in Figure 8, allowing the use of the water amount inside the fiber to be used as internal calibration for the amount of hydrogen peroxide [39]. The ratio of the peak intensities is calculated throughout the measurement at each sampling interval and the average value of the Raman ratio is plotted in Figure 8 against the known sample concentration, with vertical error bars indicating the standard deviation of each ratio. The observed linear relationship between the Raman intensities ratio and the hydrogen peroxide concentration allows the use of this internal calibration technique to add quantitative information to the measurements in a way that does not depend on input laser power fluctuations, coupling instabilities and other changes in the fiber environment.

**Figure 9.**
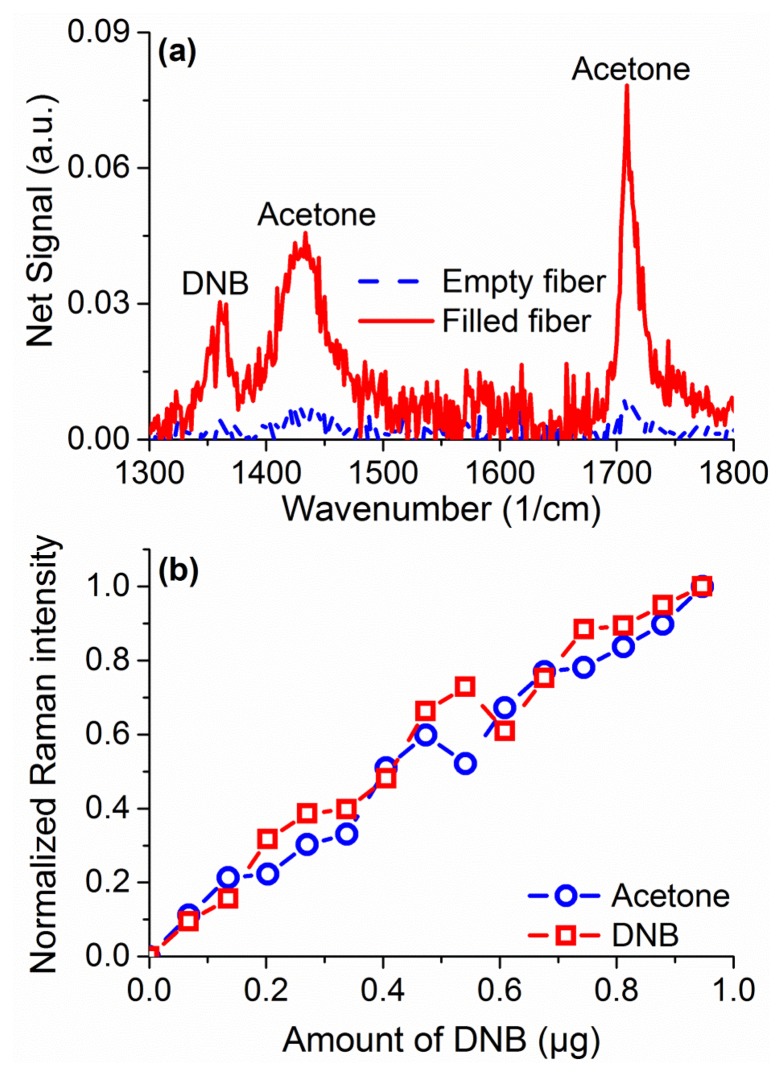
(**a**) Comparison of Raman spectra collected from an empty fiber (dashed line) and a fiber filled with a 2% w.t. 1,4-dinitrobenzene (DNB) acetone solution after subtraction of the silica background signal using a suspended core fiber; (**b**) Integrated intensity for DNB (squares) and acetone (circles) Raman spectra as a function of the amount of DNB inside a suspended core fiber.

Raman sensing has the advantage of not requiring any tagging molecules (such as fluorophores) or surface modification to work across different species. As a demonstration of that in suspended core fibers, measurements were performed to sense 1,4-dinitrobenzene (DNB), a substitute molecule for 2,4,6-trinitrotoluene (TNT), dissolved in acetone at 2% w.t. In the region of 1,000 to 2,000 cm^−1^ a number of peaks are observed, seen in Figure 9a for a filled fiber in comparison to an empty one, originating in acetone and DNB [40]. The peaks at 1,362 cm^−1^ and 1,596 cm^−1^ are signature peaks for DNB, while the peaks at 1,430 and 1,710 cm^−1^ come from acetone. As for the hydrogen peroxide, quantification of DNB can be achieved by comparing the ratio of the Raman peak to the Raman peak of the solvent, in this case acetone. Quantification of DNB is shown in Figure 9b, demonstrating a linear response for sub-microgram detection quantities. These results demonstrate the ability of the suspended core Raman sensing platform to uniquely identify different explosive species without further modifications or requirements at comparable detection limits.

## 4. Conclusions

In this work we have successfully demonstrated a liquid-phase explosives sensing platform combining silica suspended core fibers with Raman spectroscopy to detect microgram amounts of explosive species in nanolitre sampling volumes. This is based on an unmodified suspended core fiber as a Raman sensing platform, making use of the relatively large power fractions of excitation light available in this geometry to interact with analyte molecules along long lengths of the fiber. Results from numerical modeling that includes higher order excitation and Raman optical modes within the fiber to study the effect of the core radius on the signal-to-background ratio compare well against experimental observations, guiding the choice of a small core suspended core fiber to optimize the fiber's sensing performance. Using silica suspended core fibers with a 0.85 μm core radius, sub-microgram quantities of hydrogen peroxide in 60 nL sampling volumes of aqueous solution are identified on the basis of their unique Raman fingerprint. In addition, by using the Raman signature of water as an internal calibration standard, quantification of the hydrogen peroxide content is possible. The same system can be used without any further modifications to detect 1,4-dinitrobenzene, a member of the nitroaromatic explosives group, at similar concentrations. These results highlight the potential for small volume, real time identification, and quantification of explosives in solutions by using suspended core fibers as active dip sensor elements.
